# Expression of apoptosis regulatory proteins of the Bcl-2 family and p53 in primary resected non-small-cell lung cancer

**DOI:** 10.1038/sj.bjc.6690152

**Published:** 1999-02

**Authors:** M M Borner, P Brousset, B Pfanner-Meyer, M Bacchi, S Vonlanthen, M A Hotz, H J Altermatt, D Schlaifer, J C Reed, D C Betticher

**Affiliations:** University of Bern, Bern, 3010, Switzerland; Laboratoire d'Anatomie-Pathologique, Hôpital de Purpan, 31059 Toulouse Cedex, France;; The Swiss Institute for Applied Cancer Research, Bern, 3008, Switzerland; The Burnham Institute, La Jolla, CA, 92037, USA

**Keywords:** non-small-cell lung cancer, p53, Bcl-2, Mcl-1, Bax, Bak

## Abstract

Proteins of the Bcl-2 family as well as p53 are important regulators of apoptosis. Alterations in the expression of these proteins can contribute to the formation of cancer, as well as influence tumour response to chemo- and radiotherapy. We used antibodies specific for the human Bcl-2, Mcl-1, Bax, Bak and p53 proteins to examine the expression of these apoptosis-regulating genes in 49 archival specimens of patients with radically resected non-small-cell lung cancer (NSCLC). Tumour cells containing immunostaining for the antiapoptotic proteins Bcl-2 and Mcl-1 were present in 31% and 58% of the cases evaluated, respectively, whereas immunopositivity for the proapoptotic proteins Bax and Bak was found in 47% and 58% of the samples. p53 immunopositivity was detected in 61% of the samples. The expression of Bcl-2 and p53 and the expression of Mcl-1 and Bax showed a positive association (*P*= 0.02 and *P*= 0.06 respectively), whereas the expression of Bax was inversely related to p53 (*P*= 0.008). The expression of Bcl-2 had a negative influence on relapse-free survival in this population of primary resected NSCLC patients (*P*= 0.02). The expression of p53 and Bcl-2 was significantly associated with metastasis-free survival (*P*< 0.01). Only patients with p53-positive tumours developed metastases during the follow-up period. Our results establish the frequent expression of the Bcl-2 family proteins Bcl-2, Mcl-1, Bax and Bak in NSCLC. It can be expected that Bcl-2 family members have no straightforward impact on clinical outcome in this disease because their interactions in the regulation of apoptosis are complex. © 1999 Cancer Research Campaign


*British Journal of Cancer* (1999) **79**(5/6), 952–958
© 1999 Cancer Research Campaign
Article no. bjoc.1998.0152

### Expression of apoptosis regulatory proteins of the Bcl-2 family and p53 in primary resected non-small-cell lung cancer

**MM Borner[1], P Brousset[2], B Pfanner-Meyer[1], M Bacchi[3], S Vonlanthen[1], MA Hotz[1], HJ Altermatt[1], D Schlaifer[2], JC Reed[4] and DC Betticher[1]**

[1]University of Bern, 3010 Bern, Switzerland; [2]Laboratoire d'Anatomie-Pathologique, Hôpital de Purpan, 31059 Toulouse Cedex, France; [3]The Swiss Institute for Applied Cancer Research, 3008 Bern, Switzerland; [4]The Burnham Institute, La Jolla, CA 92037, USA

**Summary** Proteins of the Bcl-2 family as well as p53 are important regulators of apoptosis. Alterations in the expression of these proteins can contribute to the formation of cancer, as well as influence tumour response to chemo- and radiotherapy. We used antibodies specific for the human Bcl-2, Mcl-1, Bax, Bak and p53 proteins to examine the expression of these apoptosis-regulating genes in 49 archival specimens of patients with radically resected non-small-cell lung cancer (NSCLC). Tumour cells containing immunostaining for the antiapoptotic proteins Bcl-2 and Mcl-1 were present in 31% and 58% of the cases evaluated, respectively, whereas immunopositivity for the proapoptotic proteins Bax and Bak was found in 47% and 58% of the samples. p53 immunopositivity was detected in 61% of the samples. The expression of Bcl-2 and p53 and the expression of Mcl-1 and Bax showed a positive association ($P = 0.02$ and $P = 0.06$ respectively), whereas the expression of Bax was inversely related to p53 ($P = 0.008$). The expression of Bcl-2 had a negative influence on relapse-free survival in this population of primary resected NSCLC patients ($P = 0.02$). The expression of p53 and Bcl-2 was significantly associated with metastasis-free survival ($P < 0.01$). Only patients with p53-positive tumours developed metastases during the follow-up period. Our results establish the frequent expression of the Bcl-2 family proteins Bcl-2, Mcl-1, Bax and Bak in NSCLC. It can be expected that Bcl-2 family members have no straightforward impact on clinical outcome in this disease because their interactions in the regulation of apoptosis are complex.

**Keywords:** non-small-cell lung cancer; p53; Bcl-2; Mcl-1; Bax; Bak

The activity of a large variety of genes determines the biological behaviour of an individual tumour. Because cancer can be defined as the unscheduled accumulation of cells, genes controlling growth and proliferation must be critically involved in this disease process. However, proliferation is numerically balanced by cell death in normal tissue homeostasis and only recently has deranged cell death control evolved as an important keyplayer in the pathogenesis of cancer (Fisher, 1994). Systematic studies on parameters of cell loss in human tumour samples are scarce to date.

Cells normally die by apoptosis in the healthy organism (Jacobson et al, 1997; Nagata, 1997). Apoptosis is a genetically encoded cell death programme defined by characteristic morphological and biochemical changes which reflect the fact that normal cell death must proceed without afflicting damage to neighbouring cells. Various hierarchy levels of genes are involved in the control of cell death. Sensors such as the tumour suppressor p53 can trigger apoptosis as a reaction to unfavourable conditions, such as DNA-damaging drugs or hypoxia. More downstream, the interplay of different Bcl-2 family members determines whether apoptosis is allowed to proceed or not. At the executive end of this hierarchy, a whole cascade of caspases is involved in effecting the typical morphological changes of apoptosis. The interplay of all these gene products and other factors determines the threshold for the induction of apoptosis in an individual cell (Fisher, 1994).

*Received 19 March 1998*
*Revised 5 June 1998*
*Accepted 10 July 1998*

*Correspondence to:* MM Borner, Institute of Medical Oncology, University of Bern, Inselspital, CH-3010 Bern, Switzerland

Among the regulators of cell death, Bcl-2 (B-cell lymphoma/leukaemia 2) stands out for its evolutionary conservation and its ability to regulate a very downstream step of the cell death pathway (Nagata, 1997; Reed, 1997). High levels of Bcl-2 protein can protect cancer cells from apoptotic cell death induced by a wide variety of stimuli and insults, including radiation and chemotherapeutic drugs. The discovery of genes encoding proteins with significant amino acid sequence homology has led to the expanding family of Bcl-2-related genes (Reed, 1997). The composition or ratio of the different Bcl-2 family members may play an important role in the pathogenesis of cancer and determine the chemosensitivity in an individual tumour. The molecular details of these interactions are not fully understood. Some of the Bcl-2 family members such as Mcl-1 (myeloid cell leukaemia 1) seem to block cell death, whereas others such as Bax or Bak abrogate Bcl-2 function and promote cell death (Reed, 1997). With the exception of Bcl-2, little is known about the expression of the different Bcl-2 family members in human cancer at present.

Mutations of the p53 tumour-suppressor gene belong to the most frequent gene alterations in solid tumours (Harris, 1996). Loss of p53 function may partly explain their notorious chemo- and radiotherapy resistance because DNA damage cannot be sensed and translated into apoptosis (Fisher, 1994; Borner et al, 1995; Kinzler and Vogelstein, 1996). In addition, p53 mutations can also lead to gain-of-function conferring increased tumorigenicity, proliferative capacity, metastatic potential and tissue invasiveness (Harris, 1996).

Lung cancer is the leading cause of cancer death in Europe and the USA (Boring et al, 1994). More than 150 000 new cases are diagnosed in Europe every year, but less than 10% of these patients can be cured. Eighty per cent of malignant lung tumours are non-small-cell lung cancer (NSCLC). Despite wide surgical excision, metastases and local relapse are frequent after resection of early stage NSCLC. This makes the availability of prognostic factors for these events highly desirable to guide the use of (neo)adjuvant treatment modalities and to aid the decision about the extent of the operative procedure. The present study examined the differential expression pattern and the prognostic significance of the Bcl-2 family members Bcl-2, Mcl-1, Bax and Bak and of p53 in primary resected NSCLC.

### PATIENTS AND METHODS

### Patient features

Tumour samples were obtained from 49 consecutive patients (three women, 46 men) who underwent complete surgical resection for NSCLC at the Division of Thoracic Surgery of the University Hospital of Bern, Switzerland.

### Immunohistochemistry

Paraffin-embedded tumour specimens that had been fixed in neutral-buffered formalin were sectioned (4 μm) and immunostained using anti-peptide polyclonal antisera specific for Mcl-1 (1 : 800), Bax (1 : 2000) and Bak (1 : 1000), as previously described in detail (Krajewska et al, 1996). The specificity of all these antibody reagents had been demonstrated based on comparisons with preimmune serum, peptide competition experiments, and immunoblot analyses of tissue extracts and cell lines (Krajewska et al, 1996). Monoclonal antibodies (both from Dako, Glostrop, Denmark) were used for p53 (M 7001, Dako-p53, DO-7; 1 : 50) and for Bcl-2 (MO 887, Dako-Bcl-2, 124; 1 : 50). Immunodetection was achieved by an avidin–biotin horseradish-peroxidase-based colourimetric method using 3,3′-diaminobenzidine as the chromogen, followed by light counterstaining with haematoxylin. The slides were assessed for each antibody by two reviewers unaware of patients characteristics and outcome. Specimens were considered immunopositive when > 50% of the tumour cells had clear evidence of immunostaining. Negative controls were processed by exclusion of the primary antibody. The positive internal controls are described in the Results section.

### Statistical analyses

Association of immunostaining results (p53, Bcl-2, Mcl-1, Bax, Bak) with other categorical features (tumour histology, stage, differentiation, lymphocytic infiltration, necrosis, smoking history) was tested using the Fisher's Exact test. Actuarial relapse-free survival rates were calculated from the date of the definitive tumour operation to local relapse or the occurrence of metastases. Complete follow-up information was available in 47 patients. Kaplan–Meier estimates were used for relapse-free distributions (Kaplan and Meier, 1958). Differences between groups were evaluated with the log-rank test. All tests were two-sided.

### RESULTS

A total of 49 samples of primary resected NSCLC were processed for p53, Bcl-2, Mcl-1, Bax and Bak expression by immunohistochemistry. Table 1 lists the frequency of positivity for the various markers. The immunostaining for p53 was nuclear (Figure 1A), whereas staining for Bcl-2 family proteins was typically cytoplasmic, because these proteins are usually associated with cytosolic organelles such as mitochondria and endoplasmatic reticulum. Mcl-1 and Bak also showed nuclear reactivity, which is in keeping with the possible interaction of Bcl-2 family proteins with the nuclear membrane (Reed, 1997). Patient and tumour characteristics are listed in Table 2. In our series, a relatively high proportion of patients with squamous cell carcinomas was present. Most patients were men and the median age of the study population was 64 (range 45–79) years. Additional characteristics of this series have been described before (Betticher et al, 1996).

**Table 1** Immunohistochemical analysis of the NSCLC tumour samples with antibodies against human p53, Bcl-2, Mcl-1, Bax and Bak

| | p53 | Bcl-2 | Mcl-1 | Bax | Bak |
|---|---|---|---|---|---|
| | – | – | + | – | – |
| | – | – | + | + | + |
| | – | – | – | + | + |
| | – | – | + | + | + |
| | + | – | + | – | – |
| | + | – | + | + | + |
| | + | – | + | + | – |
| | – | + | + | + | + |
| | – | – | – | – | – |
| | – | – | + | + | + |
| | + | + | + | – | – |
| | – | – | – | + | + |
| | – | + | + | + | – |
| | – | – | – | – | + |
| | – | – | + | + | + |
| | + | – | – | – | + |
| | + | – | + | – | – |
| | – | – | – | – | – |
| | + | – | + | – | + |
| | + | + | – | – | + |
| | + | + | + | – | + |
| | + | – | – | – | + |
| | + | – | + | – | + |
| | + | – | – | + | – |
| | + | + | + | + | + |
| | + | – | + | + | – |
| | + | + | – | – | – |
| | + | + | + | + | + |
| | + | – | + | + | + |
| | + | – | – | – | + |
| | + | – | + | – | + |
| | + | + | – | – | – |
| | – | – | – | + | + |
| | + | + | – | – | – |
| | + | – | – | – | + |
| | – | – | + | + | + |
| | – | – | + | + | – |
| | + | + | + | + | – |
| | – | – | – | na | – |
| | + | + | + | + | + |
| | + | – | + | – | – |
| | + | – | – | – | + |
| | + | – | + | – | – |
| | + | + | – | – | + |
| | + | + | na | na | na |
| | + | + | – | – | + |
| | – | – | + | + | – |
| | – | – | – | – | – |
| | – | – | + | + | + |
| Positive (%) | 61 | 31 | 58 | 47 | 58 |

© Cancer Research Campaign 1999

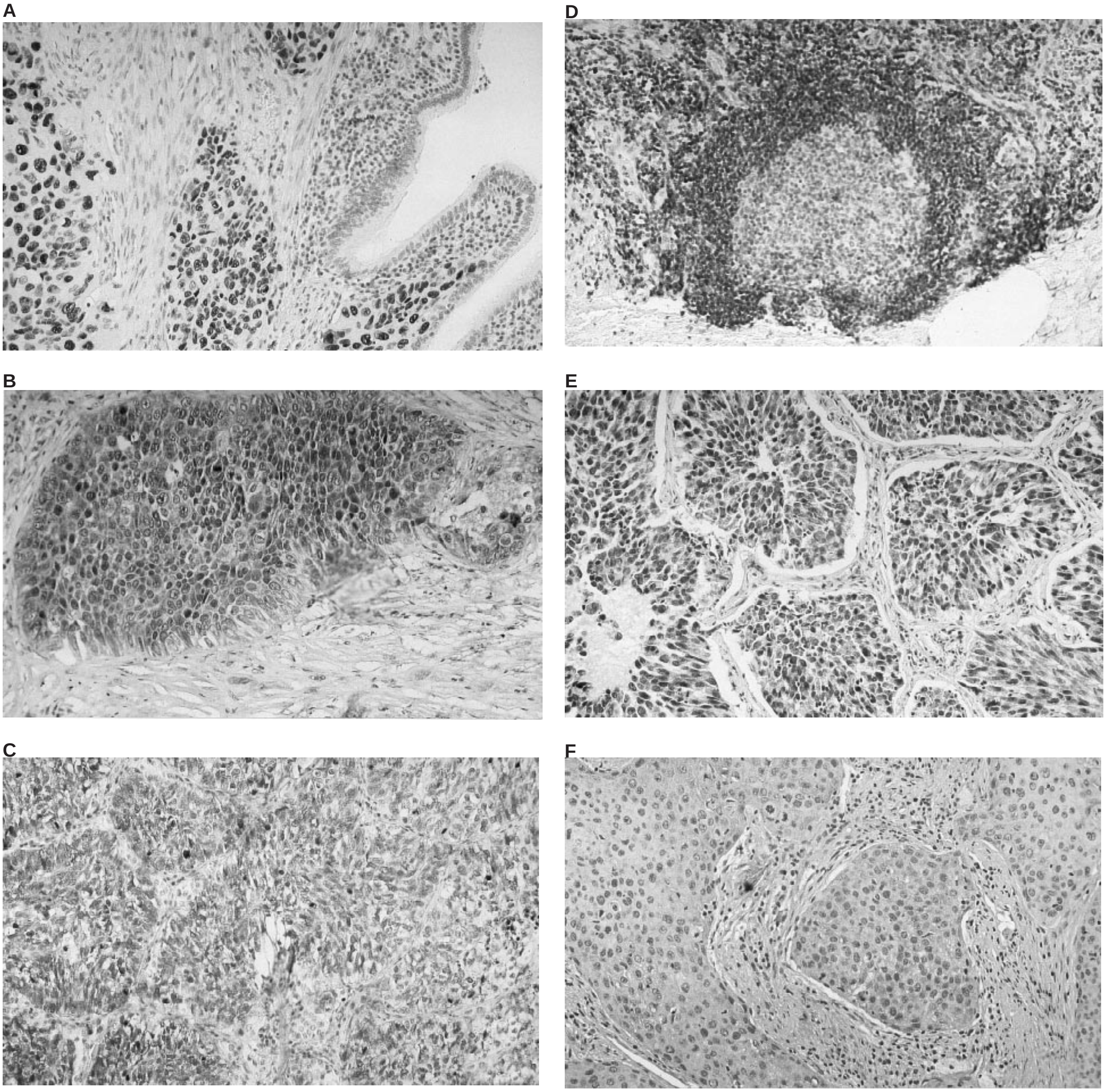

**Figure 1** Photomicrographs of representative examples of immunohistochemical staining of NSCLC with p53, Bcl-2, Mcl-1, Bax, and Bak. (**A**) Typical nuclear p53 immunostaining in a stage I squamous cell carcinoma. Bcl-2 family proteins typically show cytoplasmic staining (**B**–**F**). (**B**) Mcl-1 immunoreactivity in tumour cells of a stage III squamous cell carcinoma. (**C**) Focal Bcl-2 immunoreactivity in tumour cells of a stage I squamous cell carcinoma. (**D**) Bcl-2 immunoreactivity of mantle zone lymphocytes as a positive internal control. Note the Bcl-2-negative germinal centre cells. (**E**) Bax immunoreactivity in tumour cells of a stage I adenocarcinoma (**F**) Bak immunoreactivity in tumour cells of a stage I adenocarcinoma

### Analysis of protein expression and clinical features

### *p53*

The monoclonal antibody DO-7 specifically detects the wild-type and mutant forms of human p53. Positive immunostaining for p53 suggests mutant p53 because normal p53 is virtually undetectable by antibody-mediated staining owing to its rapid degradation (Harris, 1996). Immunoreactivity for p53 was found in 61% of the tumours (Figure 1A), which compares well with other descriptions in the literature (Apolinario et al, 1997). There was significant positive association of p53 and Bcl-2 expression ($P = 0.02$, Fisher's exact test; Table 3A), whereas p53 and Bax were inversely related ($P = 0.008$; Table 3B). There was no association of p53 expression with any of the clinicopathological features listed in Table 1. However, patients with p53 overexpression were more likely to have a history of smoking ($P = 0.05$, Fisher's exact test; Table 3C).

### *Bcl-2*

Immunoreactivity for Bcl-2 was found in 31% of the tumours (Figure 1C). The basal layers of the bronchial epithelium or mantle zone lymphocytes (Figure 1D), in which normal to strong intensity Bcl-2 immunostaining was typically seen (Pezzella et al, 1993), served as a positive control. Except for p53 (see above), there was no significant correlation between Bcl-2 accumulation and the

© Cancer Research Campaign 1999

**Table 2** Patient characteristics

| | |
|---|---|
| Number of patients | 49 |
| Sex | |
| Male | 46 |
| Female | 3 |
| Histology (WHO, 1981) | |
| Squamous carcinoma | 34 |
| Adenocarcinoma | 9 |
| Large cell carcinoma | 6 |
| Tumour differentiation | |
| Good–intermediate | 31 |
| Poor | 18 |
| Lymphocyte infiltration of the tumour | |
| Poor | 24 |
| Moderate–important | 25 |
| Tumour necrosis | |
| None–moderate | 30 |
| Important | 19 |
| Stage (UICC, 1987) | |
| I | 27 |
| II | 3 |
| III | 19 |
| Surgical intervention | |
| Lobectomy | 36 |
| Pneumonectomy | 13 |

**Table 3** Two-sided tables for Fisher's exact *t*-test calculations

| A | Bcl-2 negative | Bcl-2 positive |
|---|---|---|
| p53 negative | 17 | 2 |
| p53 positive | 17 | 13 |

| B | Bax negative | Bax positive |
|---|---|---|
| p53 negative | 5 | 13 |
| p53 positive | 20 | 9 |

| C | Non-smoker | Smoker |
|---|---|---|
| p53 negative | 3 | 8 |
| p53 positive | 0 | 29 |

| D | Necrosis (0–+) | Necrosis (++–+++) |
|---|---|---|
| Bcl-2 negative | 25 | 9 |
| Bcl-2 positive | 5 | 10 |

| E | Mcl-1 negative | Mcl-1 positive |
|---|---|---|
| Bax negative | 15 | 10 |
| Bax positive | 4 | 18 |

expression of any of the other oncoproteins of interest. Among the other clinical and pathological variables, Bcl-2 expression was significantly associated with necrotic features in the tumour ($P = 0.01$, Fisher's exact test; Table 3D).

### *Mcl-1*

Immunoreactivity for Mcl-1 was found in 58% of the tumours (Figure 1B). Chondrocytes and germinal centre lymphocytes were used as positive internal control for Mcl-1 (Krajewski et al, 1995). There was a trend for an association between Mcl-1 and Bax expression ($P = 0.06$, Fisher's exact test; Table 3E), but no correlation between Mcl-1 and other clinicopathological parameters.

### *Bax*

Immunoreactivity for Bax was found in 47% of the tumours (Figure 1E). Interfollicular lymphocytes served as an internal positive control for Bax expression (Krajewski et al, 1994). Except for the positive association with Mcl-1 and the negative association with p53 expression (see above), there was no relation between Bax expression and other clinicopathological parameters.

### *Bak*

Immunoreactivity for Bak was found in 58% of the tumours (Figure 1F). Smooth muscle cells of arteries served as an internal positive control for Bak expression (Krajewski et al, 1996). There was no correlation between Bak expression and other clinicopathological parameters.

### Impact of oncoprotein expression on tumour relapse

Relapse-free survival was taken as outcome end point in this study because too few events occurred to assess overall survival. During the follow-up period, 18 out of 47 tumours (38%) have relapsed.

**Table 4** Influence of oncoprotein expression on progression-free survival

| Marker | Event | Median survival (months) | Log-rank *P*-value |
|---|---|---|---|
| p53 +/– | All | 32/nr | ns[1] |
| | Local relapse | nr/nr | ns |
| | Metastases | 32/nr | <0.01 |
| Bcl-2 +/– | All | 8/32 | 0.02 |
| | Local relapse | nr/nr | ns |
| | Metastases | 8/nr | <0.01 |
| Mcl-1 +/– | All | nr/17 | ns |
| | Local relapse | nr/23 | ns |
| | Metastases | nr/nr | ns |
| Bax +/– | All | 32/23 | ns |
| | Local relapse | nr/23 | ns |
| | Metastases | nr/nr | ns |
| Bak +/– | All | 32/23 | ns |
| | Local relapse | nr/23 | ns |
| | Metastases | 32/nr | ns |

[1] ns, not significant ($P > 0.05$).

Table 4 describes the influence of the expression of the oncoproteins under investigation on overall relapse-free survival and progression-free survival according to relapse site. Thirteen out of 47 (28%) of the patients developed distant metastases and 13 out of 47 (28%) had a local relapse. Seven (15%) of the patients had concomitant local and distant progression. Bcl-2 expression showed a negative association with overall relapse-free survival and metastasis-free survival in this study population (log rank $P$ = 0.02 and $P < 0.01$ respectively). The expression of p53 also had a

© Cancer Research Campaign 1999

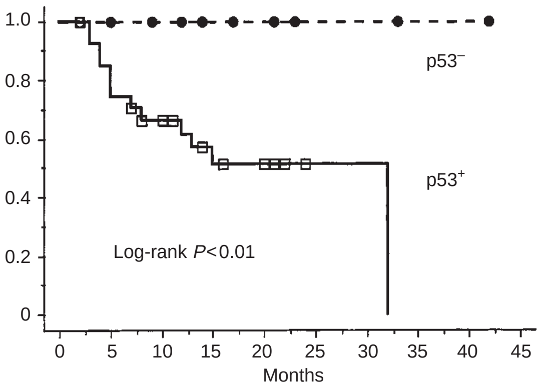

**Figure 2** Metastasis-free survival by p53

striking impact on the occurrence of metastases because no such event was observed in patients with p53-negative tumours ($P < 0.01$; Figure 2).

### DISCUSSION

We characterized here the expression of different members of the Bcl-2 gene family in primary resected NSCLC. Mcl-1, Bak, Bax and Bcl-2 were frequently expressed with 58%, 58%, 47%, and 31% of the tumours staining positive respectively. However, the percentage of immunopositive tumour cells was highly variable, implying a complex control of the production of these apoptosis-regulating proteins and suggesting the possibility that their expression is a late event in the formation of NSCLC. At the protein level, the various Bcl-2 family members can dimerize with one another, with one monomer antagonizing or enhancing the function of the other. In this way, the ratio of inhibitors to activators may determine the propensity of a cell to undergo apoptosis. With the exception of Bcl-2, relatively little is known about the expression of other family members in human cancer. In a series of 64 prostate cancer patients, Bax was expressed in all samples, whereas Mcl-1 expression was found in 81% and Bcl-2 in 25% of the cases (Krajewska et al, 1996). In stomach cancer, the antiapoptotic proteins Bcl-2 and Mcl-1 were present in 54% and 75% of the cases evaluated, whereas the proapototic proteins Bax and Bak were found in 92% and 88% of the specimens respectively (Krajewska et al, 1996). In all three series, Bcl-2 has shown the most restricted expression of all family members. Because one function of Bcl-2 is to provide protection from apoptosis, these results could be taken as evidence for a low apoptosis threshold of these tumours. However, this is contradicted by their notorious chemoresistance. Thus, it is possible that the role of Bcl-2 is redundant and that other antiapoptotic members of the Bcl-2 family can take over its function, as suggested by the reciprocal pattern of Bcl-2 and Mcl-1 expression in normal tissues (Krajewski et al, 1995). Because transformation per se seems to lower the apoptosis threshold, apoptosis-suppressing activity could be a requirement for the occurrence of cancer (Fisher, 1994). We found that the expression of Mcl-1 and Bax was positively correlated in our NSCLC samples. A similar pattern has been found in Reed–Sternberg cells of Hodgkin's disease, suggesting that Bcl-2 or other family members such as Mcl-1 may neutralize the cell death-promoting activity of Bax allowing for malignant cell survival (Brousset et al, 1996). Until more details are known about the regulation of the expression and the interaction of these proteins, it is difficult to judge on the significance of these findings.

The prognostic role of Bcl-2 in NSCLC is controversial. Studies in patients with NSCLC have demonstrated that Bcl-2 expression is associated with favourable prognosis (Pezzella et al, 1993; Fontanini et al, 1995). This finding is against mainstream thinking because the antiapoptotic action of Bcl-2 is expected to confer a survival advantage to the cancer cell. Bcl-2 has recently been shown to suppress the proliferative activity of cells, which could explain the less aggressive biology of Bcl-2-positive tumours (Borner, 1996). However, Pastorino et al (1997) did not find Bcl-2 serving as a prognostic factor for overall survival in their series of patients with stage I lung cancer. Although patients with Bcl-2-positive tumours had a better prognosis in their univariate analysis for time to recurrence, this factor dropped out in the multivariate analysis of this large series of 515 cases. Brambilla et al (1996) examined Bcl-2 expression in 121 neuroendocrine lung tumours and found a shortened survival in patients with Bcl-2-positive tumours. These results underline the fact that different Bcl-2 family proteins and other factors contribute to the biology of an individual tumour, and that it may therefore not be possible to extract reliable prognostic significance from the analysis of a single member of the Bcl-2 protein family.

Unexpectedly in the context of the putative cell-death-suppressing role of Bcl-2, we found that Bcl-2-positive tumours exhibit more necrosis than Bcl-2-negative tumours. This confirms the trend described in the paper by Tormanen et al (1995), in which tumour necrosis, but not apoptosis, seemed to correlate with Bcl-2 positivity in NSCLC tumours. However, the same authors have found a positive association between the extent of apoptosis and Bcl-2 immunoreactivity in small-cell lung cancer (Eerola et al, 1997). These results suggest independent regulatory pathways of apoptosis and necrosis in lung cancer, and could also partially reflect the inherent methodological problems in identifying and quantifying apoptotic cells in tumour samples [Potten et al, 1996]. In addition, considering the various possible consequences of Bcl-2 activity on cell cycle and cell death, it will be difficult to predict the prognostic impact of Bcl-2 expression in solid tumours.

Another key player for the role of apoptosis in the pathogenesis and treatment of cancer is the p53 tumour-suppressor gene. Abnormalities of p53 function are among the most frequently identified genetic alterations in human neoplasms, including lung carcinoma (Hollstein et al, 1991; Harris and Hollstein, 1993). p53 overexpression was present in 61% of our samples, which is in agreement with most of the published series (Chiba et al, 1990; Apolinario et al, 1997). Of all the clinicopathological features, we found p53 only to be correlated with a positive smoking history. Smoking has been described as favouring GC to AT transitions in the p53 gene in squamous cell carcinomas of the lung (Liloglou et al, 1997).

The relationship between p53 expression and prognosis in NSCLC is controversial. Nuclear p53 expression has been linked to poor prognosis (Quinlan et al, 1992; Fujino et al, 1995; Harpole et al, 1995), better prognosis (Lee et al, 1995), or no difference at all (Brambilla et al, 1993; Pastorino et al, 1997). These controversial results are difficult to interpret. Possible variables among the series were the use of different antibodies and methods, different fixation procedures, and different cut-off values. In addition, the correlation between p53 sequence and nuclear accumulation of p53 protein is indirect. Lack of accumulation may be a result of the type of mutation, such as nonsense mutations, and the presence of

 © Cancer Research Campaign 1999

accumulation may be for reasons other than mutations, which increase the half-life of p53 protein. We found a highly significant impact of p53 expression on the risk of metastatic recurrence, but not on the occurrence of local relapse. Because loss of p53 function helps cells to adapt to adverse conditions in the microenvironment, p53 mutations might confer a survival advantage to micrometastases at distant organ sites.

Aside from its interplay in the regulation of apoptosis, p53 has been shown to directly affect Bcl-2 and Bax expression. Negative response elements for wild-type p53, but not mutant p53, have been found in the 5′ untranslated regions of the *Bcl-2* gene, through which wild-type but not mutant p53 is able to mediate repression of the *Bcl-2* gene (Miyashita et al, 1994). In addition, wild-type p53 was found to strongly transactivate the expression of Bax, and p53 binding sites were also identified in the Bax promoter [Miyashita and Reed, 1995]. This has led to the speculation that Bax protein is one of the effectors of p53-induced apoptosis. We found a correlation of p53 positivity with Bcl-2 expression in our series. Because positive p53 immunostaining is mostly due to mutations, the repression of Bcl-2 expression by p53 should be lifted in these cases. Although our finding is corroborated by others (Brambilla et al, 1996), an inverse relationship between p53 and Bcl-2 expression has also been observed in NSCLC (Silvestrini et al, 1994; Fontanini et al, 1995). At the functional level, the significance of the positive association of p53 and Bcl-2 is strengthened by the inversely correlated expression of p53 and Bax in our series.

To date, pathological staging remains the most reliable determinant of prognosis in NSCLC, and the main factor in the choice of curative treatment. However, it is of great interest to verify new biological markers to define the risk of relapse or to decide on the use of (neo)adjuvant treatment. In this context, our observation of an association between the p53 and Bcl-2 status and the occurrence of distant metastases in NSCLC is of potential interest and requires confirmation.

### ACKNOWLEDGEMENTS

This research was supported by grants from the Swiss Cancer League, the Bernische Stiftung für klinische Krebsforschung, a donation from the Berner Männerchor, and the Grant in Aid Fund of the University of Bern.

### REFERENCES

Apolinario RM, van der Valk P, de Jong J, Deville W, van Ark-Otte J and Dingemans AM (1997) Prognostic value of the expression of p53, bcl-2, and bax oncoproteins, and neovascularization in patients with radically resected non-small-cell lung cancer. *J Clin Oncol* **15**: 2456–2466

Betticher DC, Heighway J, Hasleton PS, Altermatt HJ, Ryder WD, Cerny T and Thatcher N (1996) Prognostic significance of CCND1 (cyclin D1) overexpression in primary resected non-small-cell lung cancer. *Br J Cancer* **73**: 294–300

Boring CC, Squires TS, Tong T and Montgomery S (1994) Cancer statistics, 1994. *Ca Cancer J Clin* **44**: 7–26

Borner C (1996) Diminished cell proliferation associated with the death-protective activity of Bcl-2. *J Biol Chem* **271**: 12695–12698

Borner M, Myers C, Sartor O, Sei Y, Toko T, Trepel J and Schneider E (1995) Drug-induced apoptosis is not necessarily dependent on macromolecular synthesis and proliferation in the p53-negative human prostate cancer cell line PC-3. *Cancer Res* **55**: 2122–2128

Borner MM, Joncourt F and Hotz MA (1997) Similarity of apoptosis induction by 2-chlorodeoxyadenosine and cisplatin in human mononuclear blood cells. *Br J Cancer* **76**: 1448–1454

Brambilla E, Gazzeri S, Moro D, Caron, de, Fromentel C, Gouyer V, Jacrot M and Brambilla C (1993) Immunohistochemical study of p53 in human lung carcinomas. *Am J Pathol* **143**: 199–210

Brambilla E, Negoescu A, Gazzeri S, Lantuejoul S, Moro D, Brambilla C and Coll JL (1996) Apoptosis-related factors p53, Bcl2, and Bax in neuroendocrine lung tumors. *Am J Pathol* **149**: 1941–1952

Brousset P, Benharroch D, Krajewski S, Laurent G, Meggetto F, Rigal HF, Gopas J, Prinsloo I, Pris J and Delsol G (1996) Frequent expression of the cell death-inducing gene Bax in Reed–Sternberg cells of Hodgkin's disease. *Blood* **87**: 2470–2475

Chiba I, Takahashi T, Nau MM, D'Amico D, Curiel DT, Mitsudomi T, Buchhagen DL, Carbone D, Piantadosi S and Koga H (1990) Mutations in the p53 gene are frequent in primary, resected non-small cell lung cancer. Lung Cancer Study Group. *Oncogene* **5**: 1603–1610

Eerola AK, Tormanen U, Rainio P, Sormunen R, Bloigu R, Vahakangas K, Lehto VP, Soini Y and Paakko P (1997) Apoptosis in operated small cell lung carcinoma is inversely related to tumour necrosis and p53 immunoreactivity. *J Pathol* **181**: 172–177

Fisher D (1994) Apoptosis in cancer therapy: crossing the threshold. *Cell* **78**: 539–542

Fontanini G, Vignati S, Bigini D, Mussi A, Lucchi M, Angeletti CA, Basolo F and Bevilacqua G (1995) Bcl-2 protein: a prognostic factor inversely correlated to p53 in non-small-cell lung cancer. *Br J Cancer* **71**: 1003–1007

Fujino M, Dosaka AH, Harada M, Hiroumi H, Kinoshita I, Akie K and Kawakami Y (1995) Prognostic significance of p53 and ras p21 expression in non-small cell lung cancer. *Cancer* **76**: 2457–2463

Harpole DJ, Herndon 2nd J, Wolfe WG, Iglehart JD and Marks JR (1995) A prognostic model of recurrence and death in stage I non-small cell lung cancer utilizing presentation, histopathology, and oncoprotein expression. *Cancer Res* **55**: 51–56

Harris CC (1996) Structure and function of the p53 tumor suppressor gene: clues for rational cancer therapeutic strategies. *J Natl Cancer Inst* **88**: 1442–1455

Harris CC and Hollstein M (1993) Clinical implications of the p53 tumor-suppressor gene. *N Engl J Med* **329**: 1318–1327

Hollstein M, Sidransky D, Vogelstein B and Harris CC (1991) p53 mutations in human cancers. *Science* **253**: 49–53

Jacobson MD, Weil M and Raff MC (1997) Programmed cell death in animal development. *Cell* **88**: 347–354

Kaplan EL and Meier P (1958) Nonparametric estimation from incomplete observations. *J Am Stat Assoc* **53**: 457–481

Kinzler KW and Vogelstein B (1996) Life (and death) in a malignant tumour. *Nature* **379**: 19–20

Krajewska M, Fenoglio PC, Krajewski S, Song K, Macdonald JS, Stemmerman G and Reed JC (1996) Immunohistochemical analysis of Bcl-2 family proteins in adenocarcinomas of the stomach. *Am J Pathol* **149**: 1449–1457

Krajewska M, Krajewski S, Epstein JI, Shabaik A, Sauvageot J, Song K, Kitada S and Reed JC (1996) Immunohistochemical analysis of bcl-2, bax, bcl-X, and mcl-1 expression in prostate cancers. *Am J Pathol* **148**: 1567–1576

Krajewski S, Krajewska M, Shabaik A, Miyashita T, Wang HG and Reed JC (1994) Immunohistochemical determination of in vivo distribution of Bax, a dominant inhibitor of Bcl-2. *Am J Pathol* **145**: 1323–1336

Krajewski S, Bodrug S, Krajewska M, Shabaik A, Gascoyne R, Berean K and Reed JC (1995) Immunohistochemical analysis of Mcl-1 protein in human tissues. Differential regulation of Mcl-1 and Bcl-2 protein production suggests a unique role for Mcl-1 in control of programmed cell death in vivo. *Am J Pathol* **146**: 1309–1319

Krajewski S, Krajewska M and Reed JC (1996) Immunohistochemical analysis of in vivo patterns of Bak expression, a proapoptotic member of the Bcl-2 protein family. *Cancer Res* **56**: 2849–2855

Lee JS, Yoon A, Kalapurakal SK, Ro JY, Lee JJ, Tu N, Hittelman WN and Hong WK (1995) Expression of p53 oncoprotein in non-small-cell lung cancer: a favorable prognostic factor. *J Clin Oncol* **13**: 1893–1903

Liloglou T, Ross H, Prime W, Donnelly RJ, Spandidos DA, Gosney JR and Field JK (1997) p53 gene aberrations in non-small-cell lung carcinomas from a smoking population. *Br J Cancer* **75**: 1119–1124

Miyashita T, Harigai M, Hanada M and Reed JC (1994) Identification of a p53-dependent negative response element in the bcl-2 gene. *Cancer Res* **54**: 3131–3135

Pastorino U, Andreola S, Tagliabue E, Pezzella F, Incarbone M, Sozzi G, Buyse M, Menard S, Pierotti M and Rilke F (1997) Immunocytochemical markers in stage I lung cancer: relevance to prognosis. *J Clin Oncol* **15**: 2858–2865

Pezzella F, Turley H, Kuzu I, Tungekar MF, Dunnill MS, Pierce CB, Harris A, Gatter KC and Mason DY (1993) bcl-2 protein in non-small-cell lung carcinoma. *N Engl J Med* **329**: 690–694

© Cancer Research Campaign 1999 

Potten C (1996) What is an apoptotic index measuring? A commentary. *Br J Cancer* **74**: 1743–1748
Quinlan DC, Davidson AG, Summers CL, Warden HE and Doshi HM (1992) Accumulation of p53 protein correlates with a poor prognosis in human lung cancer. *Cancer Res* **52**: 4828–4831
Reed JC (1997) Double identity for proteins of the Bcl-2 family. *Nature* **387**: 773–776
Silvestrini R, Veneroni S, Daidone MG, Benini E, Boracchi P, Mezzetti M, Di FG, Rilke F and Veronesi U (1994) The Bcl-2 protein: a prognostic indicator strongly related to p53 protein in lymph node-negative breast cancer patients. *J Natl Cancer Inst* **86**: 499–504
Tormanen U, Eerola AK, Rainio P, Vahakangas K, Soini Y, Sormunen R, Bloigu R, Lehto VP and Paakko P (1995) Enhanced apoptosis predicts shortened survival in non-small cell lung carcinoma. *Cancer Res* **55**: 5595–5602
UICC American Joint Committee on Cancer (1987). *Manual for Staging of Cancer*, 3rd edn. JB Lippincott: Philadelphia
WHO (1981) *Histological Typing of Lung Tumours*, 2nd edn. World Health Organization: Geneva

*© Cancer Research Campaign 1999*
